# Irritable Hip as the Inaugural Symptom for Neuroblastoma

**DOI:** 10.1155/2018/7428350

**Published:** 2018-06-28

**Authors:** Sara Alves da Silva, Jorge Mendes, João Carvalho, Hélder Nogueira, Helena Barroca, Margarida Fernandes, Edite Tomás

**Affiliations:** ^1^Orthopedics and Trauma Department, Centro Hospitalar Tâmega e Sousa, Guilhufe, Portugal; ^2^Biopathology Department, Centro Hospitalar São João, Porto, Portugal; ^3^Pediatrics Department, Centro Hospitalar Tâmega e Sousa, Guilhufe, Portugal

## Abstract

**Case Report:**

A four-year-old girl presented with fever and a painful limp in the left hip. Pain characteristics and anemia detected in the blood analyses were the first warning signs that the hip process was not standard. Although the primary suspicion was of septic arthritis, a CT scan of the abdomen revealed an adrenal neuroblastoma.

**Conclusion:**

The presenting signs of neuroblastoma are commonly atypical. About 25% of presentations are orthopedic and mimic a variety of severe orthopedic conditions. The most important clinical dilemma is distinguishing benign and self-limiting disorders from septic or malignant processes.

## 1. Introduction

Neuroblastoma is an embryonic tumor of the peripheral sympathetic nervous system and the most common extracranial solid tumor of childhood, accounting for 8% of childhood malignancies [1, 2]. It has an annual incidence of 6.5 to 10.5 new cases per 100,000 children, and 80% present before the age of 5 [3, 4]. Signs and symptoms depend on the clinical presentation and functional status of the tumor [3, 5]. In 65% of the cases, the primary tumor is located in the abdomen, wherein the adrenal medulla is the most prevalent location [6]. Metastatic neuroblastoma is a first presentation in 60% to 75% of cases [3, 7, 8]. About 25% of presentations are orthopedic, ranging from a limp to lower limb paralysis, and can mimic a variety of severe orthopedic conditions [1, 9, 10]. It is important that these rare tumors are recognized as early as possible if treatment is to be at all effective. This condition should be borne in mind when unexplained signs and symptoms persist in children and when the disease original diagnosis fails to respond to standard therapy [11]. The classic clinical presentation of neuroblastoma is well recognized by physicians; however, we should be aware of atypical manifestations as a prompt diagnosis may help to increase survival rates and minimize irreversible damage, especially to the neural system [6]. This case highlights the importance of considering malignancy in the differential diagnosis of childhood hip pain, despite its rarity [3].

## 2. Case Report

**Figure 1:**
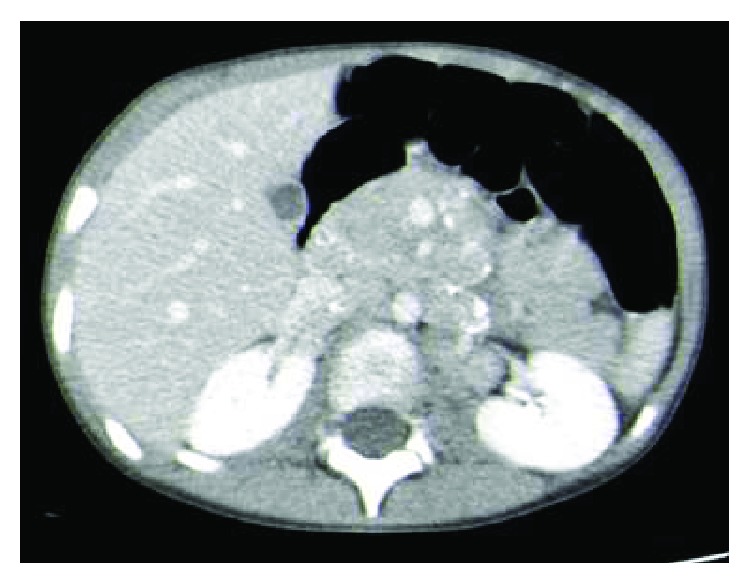
CT scan coronal cut.

A four-year-old girl presented to the emergency service with painful left hip and fever. There was no previous relevant medical history. There were no other local or systemic symptoms, except for a cervical adenopathy. On physical examination, she walked with a limp, and movements of the left hip were painful (mainly external rotation), but not restricted. Blood exam revealed anemia (Hb 8.7 gr/dL), normal WBC, ESB of 123 mm, and reactive C protein of 149.7 mg/L. An initial X-ray to the pelvis revealed no changes. An ultrasound of the left hip was performed revealing small infusion and synovitis. Guided puncture was then performed being macroscopically compatible with reactive arthritis, and general and bacteriological tests were demanded. Because of the unusual characteristics of the pain, a CT scan to the abdomen and pelvis was performed revealing a left adrenal mass and retroperitoneal adenopathies in the celiac trunk and superior mesenteric artery (Figure 1).

**Figure 2:**
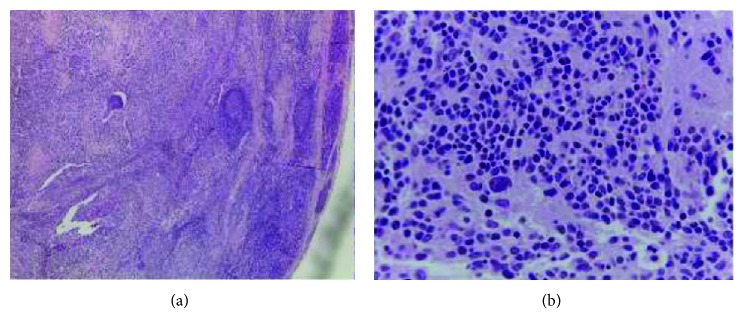
Neuroblastoma NOS: (a) neuroblastoma neuropil rich (HE, 400x), (b) lymph node partially metastasized by a neuroblastoma (HE, 40x).

Despite the painful complaints of the patient, no bone or articular involvement was found in the CT scan. No further alterations were reported in the thoracic CT scan or in peripheral blood smears. Bacteriological examination of the hip effusion was negative. MRI was also performed. The direct myelogram was compatible with infiltration from neuroblastoma. Bone marrow biopsy and cervical adenopathy specimens were collected to perform histological diagnosis. Skeletal scintigraphy demonstrated numerous points of osteoblastic activity compatible with metastatic activity, and the 12 iodine-123 metaiodobenzylguanidine scintigraphy concluded the following: “Abdominal mass with low expression of noradrenergic transporters. Diffuse bone metastasization with high expression of noradrenergic transporters. No other soft tissue involvement was detected.” In the histological report of the cervical adenopathy, the diagnosis of neuroblastoma NOS was performed. Immunohistochemistry revealed extensive expression for synaptophysin and CD56 (NCAM) and absence of expression of myogenin. (Figure 2).

Bone marrow biopsy revealed extensive metastatic involvement. The patient started chemotherapy two weeks after admission, with 8 cycles of rapid COJEC protocol. After six months of follow-up, the primary tumor was still without criteria for resection, despite a decrease in the metastatic involvement. Given the chemotherapy-related renal toxicity, it was decided to proceed with irinotecan in combination with temozolomide (TEMIRI). After thirteen months of follow-up, no significant regression of the primary tumor occurred, so surgery was contraindicated and the patient was proposed for stem cell treatment.

## 3. Discussion

Hip pain is not uncommon in childhood [2]. The most important clinical dilemma is to distinguish benign and self-limiting disorders, such as transient synovitis, from those that cause significant morbidity and mortality such as septic or malignant processes [3]. The presenting signs and symptoms of neuroblastoma are related to the site of the primary tumor, presence of metastases, and any associated paraneoplastic syndromes [12]. Around 40% of patients present with signs and symptoms owing to localized disease, and 50% of patients present with evidence of haematogenous metastases to the cortical bone, bone marrow, liver, and nonregional lymph nodes [6]. Aston reported in his neuroblastoma series that 18.3% of patients initially presented with an orthopedic complaint. The hip was the most frequent involved location and often misdiagnosed as suppurative arthritis. The hemoglobin level was the most consistent laboratory finding that suggested malignancy [13]. From the Royal Manchester Children's Hospital, four cases of metastatic neuroblastoma were reported presenting primarily with hip pain. They all posed a diagnostic dilemma with septic arthritis [10]. Mohan and Gossain also reported a case of a girl presenting with a transient synovitis of the hip later diagnosed as stage 4 neuroblastoma [2]. The mean time between disease onset and final diagnosis of malignancy was 2.5 to 3.2 months [3, 14].

Wong et al. and Huttenlocher and Newman found that a high erythrocyte sedimentation rate (ESR) (>50 mm/h) was a surprisingly good indicator of serious disease in children, particularly in patients presenting with a limp [3, 15]. Severe anemia (hemoglobin level ≤ 77 g/L) is also a warning data that can help to distinguish a malignant process from septic arthritis of the hip [13]. Aston suggested that a febrile child who presented with irritable hip should undergo ultrasonography and guided puncture to rule out infection, and, if in the presence of anemia and an ESR greater than 80 mm/h, bone marrow aspiration should be performed [13]. Parmar et al. and Trapani et al. showed that the simultaneous presence of high ESR and lactic dehydrogenase (LDH) levels or reactive C protein levels in children should lead to additional investigations to exclude malignancy [10, 14]. With respect to bone metastasis, metastatic neuroblastoma shows preference for the axial skeleton and proximal portions of the appendicular skeleton (60% of cases) [3, 6, 16]. Skeletal lesions in long bones may present radiographically as an osteolytic focus with or without periosteal reaction, a lucent horizontal metaphyseal line, or vertical linear radiolucent streaks in the metadiaphysis. Early skeletal lesions may be missed when cortical destruction is limited [6]. Plain radiographs are insensitive to destruction of less than 30% of the bone matrix, and therefore often fail to detect a bone [3, 17]. As metastatic neuroblastoma has a preference for cortical bone and bone marrow, skeletal scintigraphy with a technetium 99 bone scan is an essential part of the initial evaluation [3, 18]. Skeletal scintigraphy demonstrates sites of metastatic disease more accurately than plain radiography and also helps to differentiate osteomyelitis from metastatic neuroblastoma [3, 17]. In children younger than 1 year, however, bone scanning may show so much activity that it is difficult to detect small lesions so it is recommended that skeletal surveys be performed instead of, or in addition to, a technetium 99 bone scan [3, 19]. Iodine-123 metaiodobenzylguanidine scintigraphy is also a sensitive test for bone marrow metastases [3]. MRI is more sensitive for detection of bone lesion but the findings might be nonspecific and false negative cases are described [6]. Mohan and Gossain and White et al. in a prospective study found a higher accuracy of MRI to diagnose malignancy in relation to ultrasonography [2, 20]. Mohan and Gossain and Ranner et al. concluded that MRI was more accurate than other techniques [2, 21]. Mohan and Gossain and Lee et al. reported that MRI signal intensity was a good screening tool in children with hip pain, being useful in the differential diagnosis between septic and transient synovitis [2, 22].

## 4. Conclusion

In childhood, neuroblastoma often involves the musculoskeletal system and mimics a variety of orthopedic problems at presentation. The history and physical examination can be misleading and haematological and X-ray investigations are usually inconclusive [3]. This case emphasizes not only the need for the careful evaluation of children with persistent or atypical hip pain but also the enrichment in knowledge and the advantage of sharing experiences [1]. Despite being a rare presentation of neuroblastoma, the delay in the diagnosis may have devastating consequences. The characteristics of the pain were the first warning signs of an unusual presentation of septic arthritis. The laboratory studies further sustained the doubt because of the disproportionate degree of anemia. The unilateral presence of fluid and pain in the affected hip may be explained by the micrometastatic involvement of the bone and its eventual proximity to the articulation line with the activation of systemic inflammatory cascades, as well as some impingement in the psoas muscle as the original tumor was located near its origin. Apart from a clinically suspicious presentation, the presence of anemia raised LDH and ESR, and bone scan findings should alert the clinician to raise to other differential diagnosis, namely, neuroblastoma, as the cause of hip pain [3].
